# Cis-eQTL Analysis and Functional Validation of Candidate Genes for Carcass Yield Traits in Beef Cattle

**DOI:** 10.3390/ijms232315055

**Published:** 2022-12-01

**Authors:** Tianzhen Wang, Qunhao Niu, Tianliu Zhang, Xu Zheng, Haipeng Li, Xue Gao, Yan Chen, Huijiang Gao, Lupei Zhang, George E. Liu, Junya Li, Lingyang Xu

**Affiliations:** 1 Institute of Animal Science, Chinese Academy of Agricultural Sciences, Beijing 100193, China; 2 Animal Genomics and Improvement Laboratory, United States Department of Agriculture-Agricultural Research Services, Beltsville, MD 20705, USA

**Keywords:** cis-eQTL, GWAS, carcass yield traits, *PON3*, *PRIM2*, beef cattle

## Abstract

Carcass yield traits are of considerable economic importance for farm animals, which act as a major contributor to the world’s food supply. Genome-wide association studies (GWASs) have identified many genetic variants associated with carcass yield traits in beef cattle. However, their functions are not effectively illustrated. In this study, we performed an integrative analysis of gene-based GWAS with expression quantitative trait locus (eQTL) analysis to detect candidate genes for carcass yield traits and validate their effects on bovine skeletal muscle satellite cells (BSCs). The gene-based GWAS and cis-eQTL analysis revealed 1780 GWAS and 1538 cis-expression genes. Among them, we identified 153 shared genes that may play important roles in carcass yield traits. Notably, the identified cis-eQTLs of *PON3* and *PRIM2* were significantly (*p* < 0.001) enriched in previous GWAS loci for carcass traits. Furthermore, overexpression of *PON3* and *PRIM2* promoted the BSCs’ proliferation, increased the expression of *MYOD* and downregulated the expression of *MYOG*, which indicated that these genes may inhibit myogenic differentiation. In contrast, *PON3* and *PRIM2* were significantly downregulated during the differentiation of BSCs. These findings suggested that *PON3* and *PRIM2* may promote the proliferation of BSCs and inhibit them in the pre-differentiation stage. Our results further contribute to the understanding of the molecular mechanisms of carcass yield traits in beef cattle.

## 1. Introduction

Global food consumption is estimated to substantially increase by 2050, with significant growth in the demand for animal protein products [1]. Beef cattle provide a large proportion of the meat consumed by humans throughout the world [2]. Meat production is critical to profitability since it directly influences the beef industry, with great economic value [3,4,5].

GWAS is a powerful analysis method that allows for the identification of genomic regions associated with diverse phenotypes and helps us to understand the genetic architecture of complex traits [6,7,8,9]. Many studies have extensively investigated carcass yield traits in pigs, cattle and other farm animals, which have highlighted their polygenic nature [10,11,12,13,14,15]. In cattle, a previous study carried out pathway-based GWAS and revealed that the candidate genes with the GABAergic synapse pathway were biologically applicable to meat production traits in beef cattle [16]. More candidate variants associated with carcass traits were detected by using single-trait and multi-trait GWAS [17] and a single-step Bayesian regression approach [18]. In addition, Rafter et al. [19] performed CNV- and SNP-based GWAS for carcass traits in multiple breeds and identified several candidate CNVs and SNPs.

Moreover, increasing evidence suggests that SNPs associated with complex traits in farm animals are likely to be the expression of quantitative trait loci (eQTL). Incorporating eQTL information has the potential to increase the power of GWAS [14,20] and this strategy was widely used to locate candidate variants [21,22,23,24]. Moreover, eQTL analysis can enable the investigation of the genotype effect on gene expression levels and the interpretation of GWAS, which offers promise for characterizing functional sequence variation and understanding the basic processes of gene regulation [25]. For instance, a recent study reported that regulatory variant sets, including gene expression QTLs (geQTLs), splicing QTLs (sQTLs) and allele-specific expression QTLs (aseQTLs) discovered from multiple tissues, have regulatory and evolutionary effects for complex traits in dairy cattle [26]. Another analysis identified that genes including *PDE8B*, *NTF3*, *ZNF445* and *OR4S1* were eQTL master regulators associated with a large proportion of meat-quality-related DEG genes in beef cattle [27]. A previous study also found six QTL regions associated with residual food intake (RFI) in the Angus population, five of which contained genes that have expression associated with RFI [28]. A recent study conducted a colocalization analysis of cis-eQTLs and GWAS loci based on the Cattle Genotype Tissue Expression (CattleGTEx) atlas, and they found that the top GWAS associations of traits were significantly enriched for regulatory QTLs in their relevant tissues [29].

Although comprehensive analyses of candidate genes using both GWAS and eQTL analysis have been undertaken, functional validation of these genes is still rare. Thus, the objectives of this study were to (1) detect candidate genes using both gene-based GWAS and eQTL analyses for carcass yield traits; (2) carry out the comparative analysis of candidate genes using the colocalization method; and (3) perform the functional validation for candidate genes.

## 2. Results

### 2.1. Summary Statistics of Sequencing Dataset

A total of 2.78 billion raw reads from *longissimus dorsi* tissues were generated by RNA sequencing. The total size was 833.89 Gb, and the average sequencing size was 6.95 Gb, ranging from 5.88 to 8.89 Gb. After strict quality control, a total of 796.03 Gb of high-quality reads were mapped to the reference genome (ARS-UCD1.2). The average mapping rate of samples was 95.47%, ranging from 92.32 to 97.33% (Table S1).

### 2.2. Gene-Based GWAS

**Figure 1:**
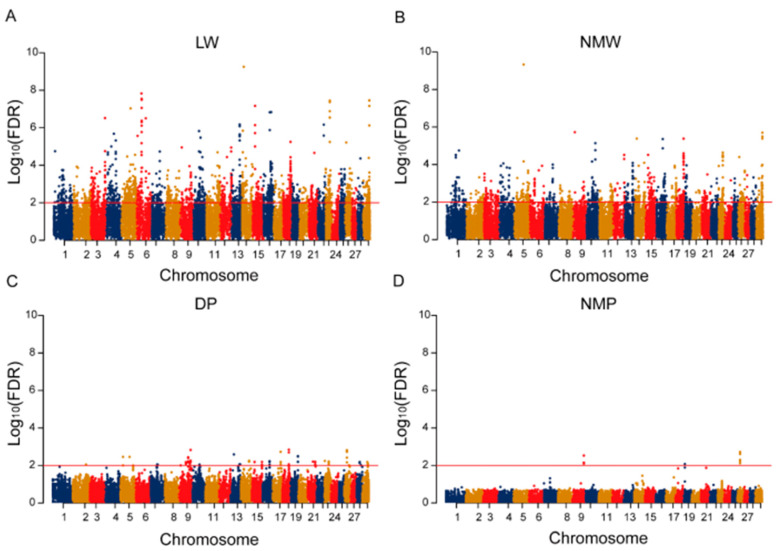
(**A**–**D**) The Manhattan plots of the gene-based GWAS results for live weight (LW), net meat weight (NMW), dressing percentage (DP) and net meat percentage (NMP). Chromosomes are shown in different colors.

To explore the association between genes and carcass yield traits (LW, NMW, DP, NMP), we performed gene-based GWAS based on a generalized gene set analysis of GWAS data. A total of 2196 candidate Ensembl genes were associated with LW, 1047 with NMW, 70 with DP and 9 with NMP (FDR < 0.01), and the Manhattan plots are shown in Figure 1. ENSBTAG00000021953 showed the most significant signal for NMW (FDR = 4.62 × 10^−10^). Remarkably, 805 genes were detected for both LW and NMW, and eight genes were identified for DP and NMP, suggesting their pleiotropy for meat yield traits (Table S2). GO enrichment showed that 15 terms were enriched in molecular functions, 100 terms in biological processes and 18 terms in cellular components, including protein binding (GO: 0005515, *p* = 2.79 × 10^−19^), anatomical structure development (GO: 0048856, *p* = 2.12 × 10^−13^) and the cellular developmental process (GO: 0048869, *p* = 1.70 × 10^−7^) (Table S3).

### 2.3. Cis-eQTL

**Figure 2:**
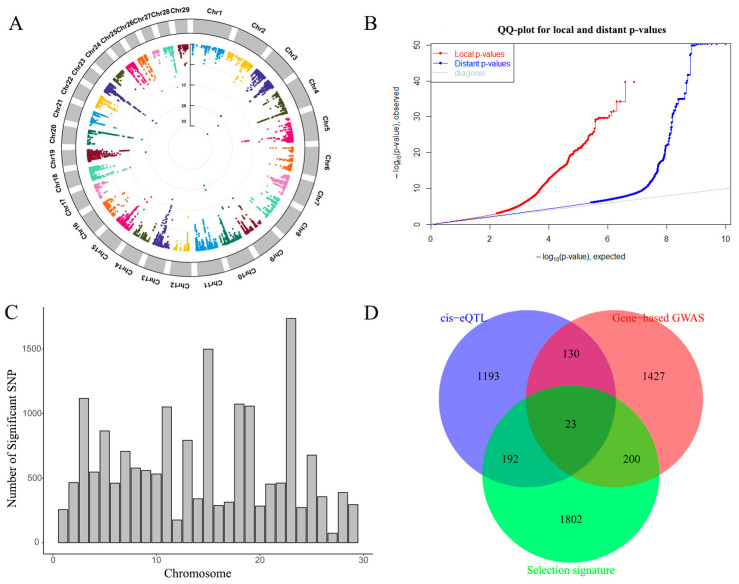
(**A**) The CMplot of eQTLs, chromosomes are shown in different colors. (**B**) The QQ plot of eQTLs. (**C**)The distribution of significant cis-eQTL SNPs in autosomes. (**D**) The Venn plot among gene sets from eQTL, gene-based GWAS and selection signature.

In this study, a total of 18,239 cis-eQTLs were identified with FDR < 0.05 (Table S4) and associated with 1837 Ensembl genes. The distribution of cis-eQTLs on autosomes and QQ plots are shown in Figure 2A,B. The distribution of significant cis-eQTL across autosomes is shown in Figure 2C, and we also found that the number of cis-eQTLs was larger than the number of tagged genes. GO enrichment showed that eight terms were enriched in molecular functions, 11 terms in biological processes and 13 terms in cellular components, including catalytic activity (GO: 0003824, *p* = 6.85 × 10^−14^), the small-molecule metabolic process (GO: 0044281, *p* = 4.24 × 10^−7^) and cytoplasm (GO: 0005737, *p* = 5.60 × 10^−22^) (Table S5).

### 2.4. Gene Overlap between Gene-Based GWAS and Cis-eQTLs

In this study, we detected 1538 cis-expression genes and 1780 genes from gene-based GWAS, respectively. A total of 153 shared genes were identified based on the intersection analysis of both methods. To further understand the function of candidate genes, we performed GO enrichment analysis using g:Profiler. We found that most genes were enriched in the cytoplasm (GO: 0005737, *p* = 0.0029) (Table S6). Based on our previous analysis, we further merged the genes from eQTLs, gene-based GWAS and selection signatures [17] (Table S7). Twenty-three shared genes were obtained among three gene sets (Figure 2D).

### 2.5. SNPs Associated with PON3 and PRIM2

**Figure 3:**
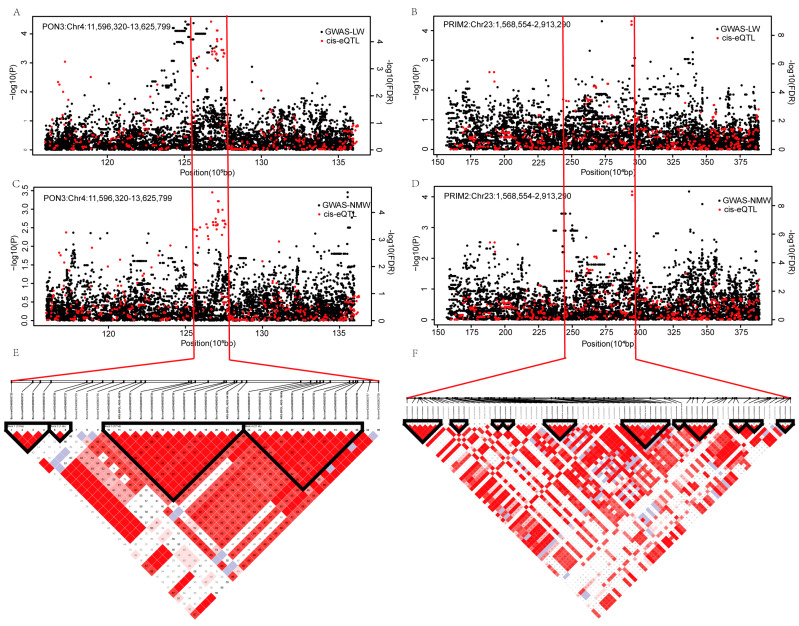
(**A**–**D**) The scatter diagram of SNPs within 1Mb upstream and downstream of the *PON3* and *PRIM2* from the previous GWAS and cis-eQTL results. Y-axis on the left side of the chart is the −log_10_(P) of the GWAS result, and on the right side is the −log_10_(FDR) of the cis-eQTL result. (**E**) The LD block from BovineHD0400003730 to BovineHD0400003759 in *PON3*. (**F**) The LD block from BovineHD2300000469 to BovineHD2300000550 in *PRIM2*.

In 153 shared genes, *PON3* (Paraoxonase 3) and *PRIM2* (DNA primase subunit 2) genes were located in a candidate QTL region for carcass yield traits, which was identified in beef cattle from the previous GWAS result [17]. The hypergeometric test identified cis-eQTLs of *PON3* and *PRIM2* as being significantly (*p* < 0.001) enriched in previous GWAS hit regions (*PON3* and *PRIM2* for LW in Figure 3A,B, *PON3* and *PRIM2* for NMW in Figure 3C,D). There were 31 and 30 significant SNPs (FDR < 0.05) associated with *PON3* and *PRIM2* in the cis-eQTL result. The most significant SNP genotypes of *PON3* and *PRIM2* were significantly correlated with the FPKM values, as shown in Figure S1. Strong linkage disequilibrium is observed around the most significant SNPs (Figure 3E,F).

### 2.6. Gene Expression Patterns in Myogenic Differentiation

**Figure 4:**
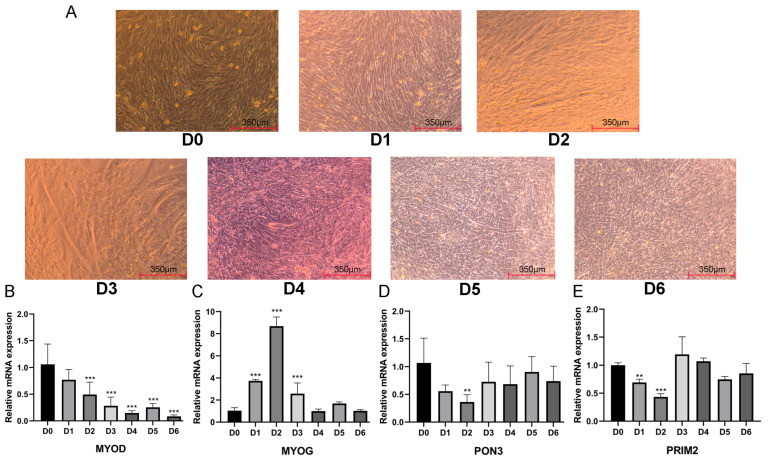
(**A**) The BSCs’ differentiation from D0 to D6. (**B**–**E**) The mRNA expression level of *MYOD*, *MYOG*, *PON3* and *PRIM2* when BSCs differentiate from D0 to D6. GAPDH was used as the reference gene. ** *p* < 0.01 and *** *p* < 0.001.

To explore the expression regulation of *PON3* and *PRIM2*, we investigated the expression levels from Day 0 (D0) to Day 6 (D6) during the myogenic differentiation of BSCs. BSCs began to aggregate on D1, and myotubes appeared on D2 and differentiated into large myotubes on D3. From D5 to D6, all cells became myotubes (Figure 4A). The expression of *MYOD* was downregulated slowly in early differentiation (D1–D2) and declined rapidly in middle differentiation (D3–D4) (Figure 4B). The expression of *MYOG* increased rapidly from the early stage of differentiation, decreased at the middle stage and returned to the normal level at the late stage (Figure 4C). The expression of *PRIM2* was downregulated in prophase differentiation and then returned to normal levels (Figure 4D). The expression of *PON3* was also downregulated in prophase differentiation and then returned to normal levels (Figure 4E).

### 2.7. Overexpression of PON3 and PRIM2 in BSCs

**Figure 5:**
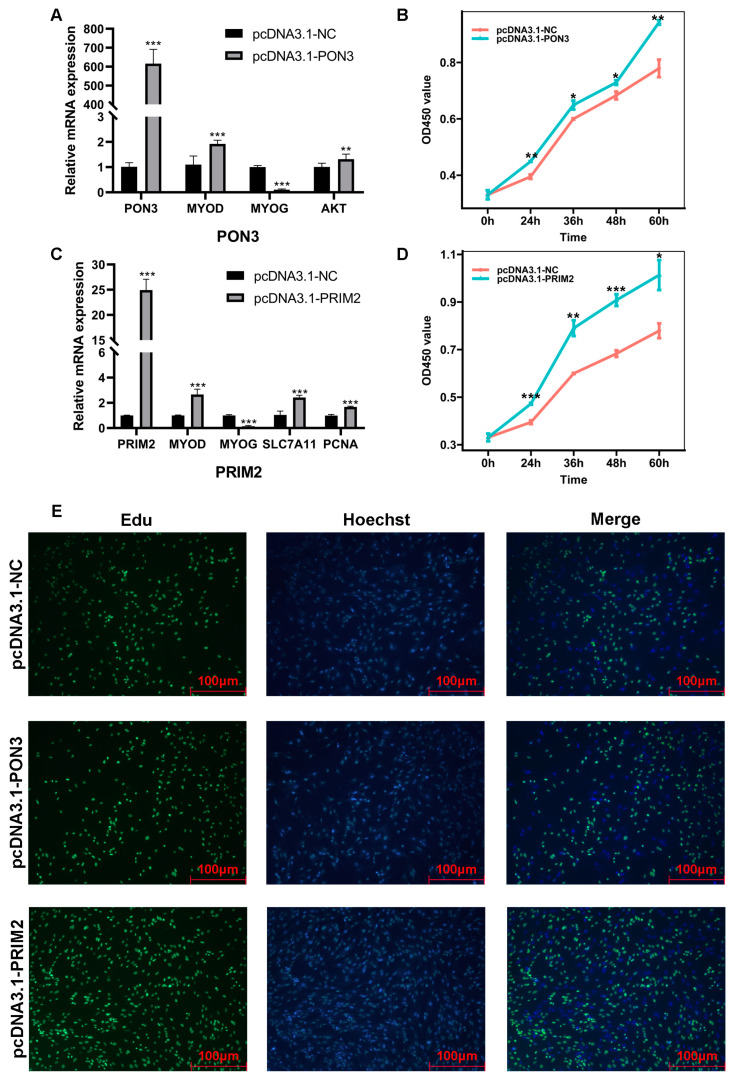
The effect of *PON3* and *PRIM2* on BSC proliferation. (**A**) The mRNA expression level of *PON3*, *MYOD*, *MYOG* and *AKT* when transfected with pcDNA3.1-PON3. *GAPDH* was used as the reference gene. (**B**) The mRNA expression level of *PRIM2*, *MYOD*, *MYOG* and *SLC7A11* when transfected with pcDNA3.1-PRIM2. *GAPDH* was used as the reference gene. (**C**) Cell Counting Kit-8 (CCK8) assay showing that overexpressed *PON3* promoted BSC proliferation. (**D**) Cell Counting Kit-8 (CCK8) assay showing that overexpressed *PRIM2* promoted BSC proliferation. (**E**) EdU staining of the BSCs after the transfection of pcDNA3.1-PON3 and pcDNA3.1-PRIM2. * *p* < 0.05, ** *p* < 0.01 and *** *p* < 0.001.

To explore the role of *PON3* and *PRIM2* in BSCs, pcDNA3.1-PON3 and pcDNA3.1-PRIM2 were constructed and used to infect BSCs. qRT-PCR analysis revealed that the mRNA expression levels of *PON3* and *PRIM2* were significantly upregulated. When the *PON3* gene was overexpressed, *MYOD* and *AKT* were significantly upregulated and *MYOG* was significantly downregulated (Figure 5A), and when the *PRIM2* gene was overexpressed, *MYOD*, *SLC7A11* and *PCNA* were significantly upregulated and *MYOG* was significantly downregulated (Figure 5B). To further explore the function of *PON3* and *PRIM2* for BSC proliferation, the CCK8 assay and EdU assay were used to analyze the proliferation of BSCs after being transfected by pcDNA3.1-PON3, pcDNA3.1-PRIM2 and pcDNA3.1-NC. The CCK8 assays showed that the overexpression of *PRIM2* or *PON3* significantly promoted BSC proliferation (Figure 5C,D). The EdU assay is shown in Figure 5E, and our results revealed that the overexpressed *PRIM2* or *PON3* significantly increased the proportion of EdU-positive cells (Figure S2). These results suggest that overexpressed *PON3* or *PRIM2* can promote BSC proliferation.

## 3. Discussion

Previous GWAS have identified many genetic variants associated with carcass yield traits in beef cattle. However, the functions of these variants on these traits are often unknown [22]. Intuitively, the genes in the closest physical proximity to top-associated variants are the most likely causal genes. Most identified genes from GWAS analysis have not yet been tested empirically because of the lack of comprehensive functional studies, and causal genes may be distinct from the nearest variants [30,31]. Thus, precise estimation of the locations and effect sizes of regulatory elements is critical to understand the relationship between genetic and phenotypic variation [32,33]. In this study, we detected several candidate genes by overlapping the genes detected from both GWAS and eQTL analyses. Finally, two top candidate genes showing strong evidence from the hypergeometric test were chosen for subsequent functional validation.

In our study, we only explored the cis-eQTL analysis. Trans-eQTL, for which the SNP is located distal to the gene (>5 Mb) or on another chromosome, usually has smaller effect sizes than cis-eQTL and thus requires larger sample sizes for detection [34]. A previous study has compared the allele-specific expression and local expression quantitative trait loci and the influence of gene expression on complex trait variation in cattle, and their findings suggested that the identified QTLs affecting beef tenderness and growth are most likely cis-eQTLs for *calpain 1*, *calpastatin* and *LCORL* [35]. In our study, using cis-eQTL and gene-based GWAS analysis, we identified 1538 cis-specific genes and 1782 GWAS genes. Among them, there were 153 genes shared by cis-eQTL and gene-based GWAS, while 23 genes were shared among cis-eQTL, gene-based GWAS and selection signatures. Among 23 genes, six genes (*SLC13A3*, *ATG7*, *ETFDH*, *GADL1*, *PRKG1* and *ACAD11*) were observed to be related to carcass yield traits, and *SLC13A3* was highlighted as a promising candidate for carcass traits in pigs [36]. In another study, increasing the expression of *ATG7* in *M. longissimus* was highly correlated with the ultrasonic *M. longissimus* area and body weight in cattle [37]. A further study indicated that the *ETFDH* expression level was different for high and low tenderness in yak meat [38] and *GADL1* was associated with beef tenderness [39]. In addition, *ACAD11* was found to be associated with meat quality (pH value, water loss rate, fat and protein and fatty acid content) [40]. *PRKG1* was reported to be a candidate gene that was differentially expressed in relation to intramuscular fat in pigs [41].

To detect the hit regions instead of exact overlaps (SNP to SNP) for the further scanning of the candidate genes, we performed a hypergeometric test using cis-eQTL and GWAS, which could increase the probability of overlap because eQTLs may be in LD with QTL. Xiang et al. [26] built functional genomic relationship matrices (GRM) using eQTL SNPs that explained a large amount of the genetic variance in 34 complex traits, including production, reproduction, management and linear assessment traits. However, our results showed only a few overlaps between cis-eQTL and single-trait GWAS SNPs, supporting two published cattle studies that showed a relatively limited overlap between eQTL and single-trait GWAS SNPs [35,42]. A more recent study also showed that correlations between local GEBV and *MGST1* expression were weak, while the peak visible around the *MGST1* gene in both the GWAS and local GEBV was clear [42]. Hence, we performed the hit region hypergeometric test with a relaxed threshold value of GWAS to capture more genes. The cis-eQTL SNPs located within 1Mb upstream and downstream of *PON3* and *PRIM2* were significantly (*p* < 0.001) enriched in GWAS hit regions. Moreover, the PON3 c.449GNA SNP is associated with longissimus dorsi lean meat deposition, suggesting that PON3 may play a role in skeletal muscle growth [43]. In previous GWAS results, *PRIM2* was associated with body size in Simmental beef cattle by single-trait analysis [44]. These results collectively suggest that *PON3* and *PRIM2* may be associated with carcass yield traits, and we carried out downstream molecular experiments to verify these two genes.

Satellite cells are muscle-derived stem cells that lie adjacent to the mature skeletal muscle fiber [45]. The asymmetrical proliferation and myogenic differentiation of satellite cells, and their fusion with existing muscle fibers, are crucial for postnatal muscle growth [46]. Thus, we transfected pcDNA-PON3 and pcDNA-*PRIM2* vectors into BSCs, and both of them can promote cell proliferation. Previous studies demonstrated that the knockdown of *PON3* decreased cell proliferation and total antioxidant capacity in ambient oxygen levels [47]. *PON3* regulates cell proliferation, cell cycle, migration and invasion in oral squamous cell carcinoma via the PI3K/Akt pathway [48]. This result was also confirmed in our study, as we found that *AKT* was significantly upregulated when we overexpressed *PON3*. In addition, *PRIM2* plays a key role in both the initiation of DNA replication and the synthesis of Okazaki fragments for the lagging strand synthesis [49,50]. In our results, overexpressed *PRIM2* upregulated the expression of *SLC7A11* and *PCNA*. Loss of *PRIM2* downregulated the expression of *SLC7A11* and β-catenin protein expression and inhibited cell proliferation [51,52]. A previous study indicated that *PRIM2* exerts its primase activity to promote DNA replication and can also induce an increase in *PCNA* expression, promote mismatch repair and promote DNA replication [53]. Notably, during BSC differentiation, *MYOD*, *PON3* and *PRIM2* were significantly downregulated, and *MYOG* was significantly upregulated, which means that *PON3* and *PRIM2* may inhibit BSCs’ myogenic differentiation. Further studies on more genes will be required using integrative methods from ‘omics’-level data sets to increase the power of detecting true causal genes, regulatory networks and pathways, in order to improve animal health, welfare and production [54].

## 4. Materials and Methods

### 4.1. Animal Sampling and Phenotype

Huaxi cattle (derived from Chinese Simmental beef cattle) with an average age of 6 months were obtained from Ulgai, Xilingol League, Inner Mongolia of China. After weaning, all individuals were moved to a fattening farm under uniform management and standardized feeding based on a total mixed ration (TMR) according to the eighth revised edition of the Nutrition Requirements of Beef Cattle (NRC, 2016) [55]. They were slaughtered at an average age of 26 months for three batches from 2020 to 2021. Blood samples were obtained with routine quarantine inspection before slaughter. *Longissimus dorsi* muscle was sampled within 10 min of slaughter at the abattoir and was flash-frozen in liquid nitrogen and stored at −80 °C until use.

Live weight (LW) was measured before slaughter. Net meat weight (NMW) was determined by subtracting the bone weight from the cold carcass weight. Dressing percentage (DP) was defined as hot carcass weight/LW ratio. Net meat percentage (NMP) was measured by NMW/cold carcass weight. In this study, farm, year and sex were considered as the fixed effects, and weight before fattening and fattening days were considered as covariates. Then, phenotype was adjusted using the general linear model and the residuals were considered as the pre-adjusted phenotype for the subsequent analysis.

### 4.2. Genotyping and Data Quality Control

Genomic DNA was extracted from blood using the DNeasy Blood & Tissue Kit (Qiagen, Valencia, CA) and stored at −20 °C. Samples were genotyped with the Illumina BovineHD BeadChip, which contains 777,962 SNPs. Before statistical analysis, the original SNP dataset was filtered using PLINK (v1.90). After quality control using the minimum allele frequency (--maf 0.05) and excluding the extraordinary chromosome (--autosome), a total of 594,005 SNPs remained for subsequent analysis.

### 4.3. RNA Preparation and Sequencing

Frozen muscle tissues were ground using the Geno/Grinder^®^ 2010 (SPEX™, Metuchen, NJ, USA). Ground tissue was homogenized in Trizol^®^ (Life Technologies™) and RNA extracted using the Trizol^®^ plus RNA extraction kit (Life Technologies™) according to the manufacturer’s instructions. RNA quality and integrity were evaluated by the 2100 Bioanalyzer (Agilent Technologies, Waldbronn, Germany). All samples with an RNA integrity number larger than 7 and a 28S/18S ratio higher than 1.0 were used for library preparation using the SureSelect Strand Specific RNA Library Prep Kit (Agilent Technologies). The cDNA library was constructed using the Illumina TruSeqTM RNA Kit (Illumina, San Diego, CA, USA) according to the manufacturer’s instructions. RNA-seq was performed on the Illumina NovaSeq 6000 platform using a pairing end strategy (read length 150 base pairs (bp)).

### 4.4. Quantification of Molecular Phenotypes

Clean paired reads were aligned to the cattle reference genome ARS-UCD1.2/bosTau9 using HISAT2 (v2.2.1) [56]. Effective reads aligned with gene regions were statistically calculated based on genomic location information specified by bovine reference genome annotations. Each Sequence Alignment Map (SAM) file was sorted and transformed into a Binary Alignment Map (BAM) file by SAMtools (1.9) [57]. Gene saturation was assessed as the number of detected genes per percent of total reads at increasing read depths, with reads sampled from each BAM file. Read counts of each gene were calculated by FeatureCounts (v2.0.0) [58]. Read counts of more than 1 in more than 30 individuals (>25%) were retained, and gene counts were transformed using the tool Variance-Stabilizing Transformation from the R package DESeq2 (version 1.20.0) [59].

### 4.5. Cis eQTL Detection

The R package MatrixEQTL was used to perform the eQTL mapping using 594,005 SNPs and 16,472 genes located on autosomes with two covariates, including the batches and age. A linear regression model was used, where the SNP genotypes were coded as 0, 1 or 2. The cis eQTL was calculated by including all variants on the same chromosome that were located within 1 Mb up- or downstream of the transcription start site or polyadenylation site of a gene locus.

### 4.6. Gene-Based GWAS

To quantify the degree of association with LW, NMW, DP and NMP for each gene, we performed the gene-based association analyses using MAGMA (v1.08) [60]. First, we extended 50kb upstream and downstream of each gene and annotated all the SNPs according to the genome position. The imputed whole-genome sequence data of 1233 individuals were obtained from a previous study, and a total of 12,102,431 variants were used for the analyses [17]. We utilized the multi-models with command “multi = snp-wise” in MAGMA software, including SNP-wise mean and SNP-wise top, which are more suitable for the genetic architectures with only a small proportion of SNPs or areas of higher LD in a gene show association. Finally, we obtained the *p*-values of all tested genes for subsequent analyses. The Benjamini and Hochberg method was adopted to correct for multiple testing, and the genes with an adjusted FDR < 0.01 were considered as the candidate genes.

### 4.7. Candidate Genes’ Annotation and Enrichment

The Ensembl Gene IDs of the candidate genes were obtained by the BioMart tool, and overlap analyses were conducted using their Ensembl Gene IDs to identify candidate genes from cis-eQTL and gene-based GWAS. A list of candidate genes was obtained by integrating the results of current and previous studies [17]. The gene set enrichment analysis for candidate genes was conducted using g:Profiler [61]. The significance of GO terms was determined by the pre-adjusted threshold (*p* < 0.05) in g:Profiler.

### 4.8. Enrichment of Cis-eQTLs in GWAS Hit Regions

From our previous GWAS results [17], we first obtained numerous significant SNPs associated with carcass yield traits. To evaluate whether a cis-eQTL was enriched with significant SNPs from GWAS, we investigated the overlap of cis-eQTLs in an interval of 17 kb up- and downstream of significant SNPs (FDR < 0.05), which was based on the mean gap size between probes on the Illumina Bovine HD. The hypergeometric test was used to investigate whether the identified cis-eQTLs were significantly enriched in GWAS hit regions [62].

### 4.9. Cell Isolation and Culture

BSCs were isolated from three cow fetal hind limb muscles based on the previously reported method [63]. The fetuses at 90 to 120 days after conception were collected immediately after removal from the uteri of slaughtered cows. The fetuses were transported to the laboratory within 2–4 h. Briefly, muscle tissue was collected in a sterile environment and shredded fully, and then type II collagenase (Gibco, Grand Island, NY, USA) and trypsin (Gibco, Waltham, MA, USA) were used to isolate cells. The isolated primary cells were cultured in a growth medium (GM) containing Dulbecco’s modified Eagle’s medium high-glucose (DMEM-H, Gibco, Grand Island, NY, USA) with 20% fetal bovine serum (FBS, Gibco, Grand Island, NY, USA) under a humidified atmosphere of 5% CO_2_ at 37 °C. The cells were passaged at 70–80% confluence and induced in the differentiation medium (DM) containing DMEM-H and 5% horse serum when reaching 100% confluence.

### 4.10. Plasmid Construction and Transfection

According to the mRNA sequence of these candidate genes in the NCBI, primers were designed using Primer Premier 5 and synthesized by Sangon (Sangon Biotech, Shanghai, China). PON3, F: ccaagctggctagttaagcttATGGGGAAGCTGATGGCAC R: ttgttcgaagggccctctagaCTAGAGCTCACAGTACAGGGCTTTG. PRIM2, F: ccaagctggctagttaagcttATGGAGTTTTCTGGAGGAAAACG, R: ttgttcgaagggccctctagaTCACTGAAGCAACTCCTCCAGTT.

The complete CDS region sequence was cloned into pcDNA3.1 using the ClonExpress MultiS One Step Cloning Kit (Vazyme Biotech Co., Jiangsu, China) and the insert was released by *HindIII* and *XbaI* digestion. All plasmids were sequenced by the Sanger sequencing method (Sangon) and were identical to the NCBI reference sequence. Then, transfection of the plasmid into BSCs was performed using lipofectamine 3000 transfection reagent (Invitrogen Life Technologies, Carlsbad, CA, USA) when the cells reached 80~90% confluence.

### 4.11. RNA Extraction, RT-PCR and qRT-PCR

The total RNA from the muscle tissues was extracted by using the Trizol (Invitrogen Carlsbad, CA, USA) method. The same amount of RNA of each sample was reverse-transcribed according to the instructions of the reverse transcription kit (TaKaRa Biotech Co., Ltd., San Jose, CA, USA). Primers for qPCR analysis are shown in Table S8, and the mRNA expression was detected using the KAPA SYBR^®^ FAST qPCR Master Mix (2X) Kit (KAPA Biosystem, Wilmington, MA, USA.) in the QuantStudio 7 Flex Real-Time PCR System (life, Carlsbad, CA, USA). The relative abundance of target mRNAs was calculated using the 2^−ΔΔCt^ method.

### 4.12. Cell Counting Kit-8 (CCK8) Assay

The cells were subcultured and seeded in 96-well plates and transfected with pcDNA3.1-PRIM2, pcDNA3.1-PON3 and pcDNA3.1-NC. Following incubation for 0 h, 24 h, 36 h, 48 h and 60 h at 37 °C, cell viability was determined by CCK-8 assays performed according to the manufacturer’s instructions. In brief, 10 µL of CCK-8 reagent (Beyotime, SH, CHN) was added to each well and cells were incubated at 37 °C in 5% CO_2_ for 2 h. Absorbance was determined at a wavelength of 450 nm with an Imark Microplate Reader (Bio-Rad, CA, USA). Each assay was performed in triplicate and repeated three times.

### 4.13. 5-Ethynyl-2′-Deoxyuridine (EdU) Assay

The cells were cultured until reaching 50% density and transfected with different plasmids. The cells were subjected to the 5-ethynyl-2′-deoxyuridine (EdU) assay 20 h after transfection, using the Cell-Light EdU Apollo488 In Vitro Kit (RiboBio, Guangzhou, China). We captured three randomly selected fields using a fluorescence microscope (TCS SP8, Leica) and determined the number of EdU-stained cells. ImageJ software was utilized to determine the percentage of EdU-positive cells.

### 4.14. Statistical Analysis

Statistical significance was determined by the following: (i) one-way analysis of variance with Tukey’s multiple comparison test was used to compare differences in mean values at the 5% significance level, and (ii) a two-sided Student’s *t*-test was utilized for the difference analysis of two contrasts. Results were visualized using GraphPad Prism 8 software (version 8.02) as mean values, with error bars representing the standard error of the mean. We considered *p* < 0.05 to be statistically significant. * *p* < 0.05; ** *p* < 0.01; *** *p* < 0.001.

## 5. Conclusions

Our study detected 1780 and 1538 genes based on gene-based GWAS and cis-eQTL analysis. Among them, we identified 153 shared genes that may play important roles in carcass yield traits. We also found that the proliferation rate of BSCs was significantly accelerated when *PON3* and *PRIM2* were overexpressed. Furthermore, *PON3* and *PRIM2* significantly decreased in expression during the pre-differentiation of BSCs. Thus, we speculate that *PON3* and *PRIM2* promote the proliferation of BSCs and inhibit their differentiation. Our results contribute to the understanding of the molecular mechanisms of carcass yield traits in beef cattle.

## Data Availability

The datasets used and analyzed during the current study are available from the corresponding author on academic request.

## Supplementary Materials

The following supporting information can be downloaded at: https://www.mdpi.com/article/10.3390/ijms232315055/s1.
