# Supplementary material for: Cis-eQTL Analysis and Functional Validation of Candidate Genes for Carcass Yield Traits in Beef Cattle
Source: Int J Mol Sci. 2022 Dec 1;23(23):15055. doi: 10.3390/ijms232315055 (PMC9736101; doi:10.3390/ijms232315055)
Supplement: Supplementary file 1 [file ijms-23-15055-s001.zip › Supplementary Figure.pdf]

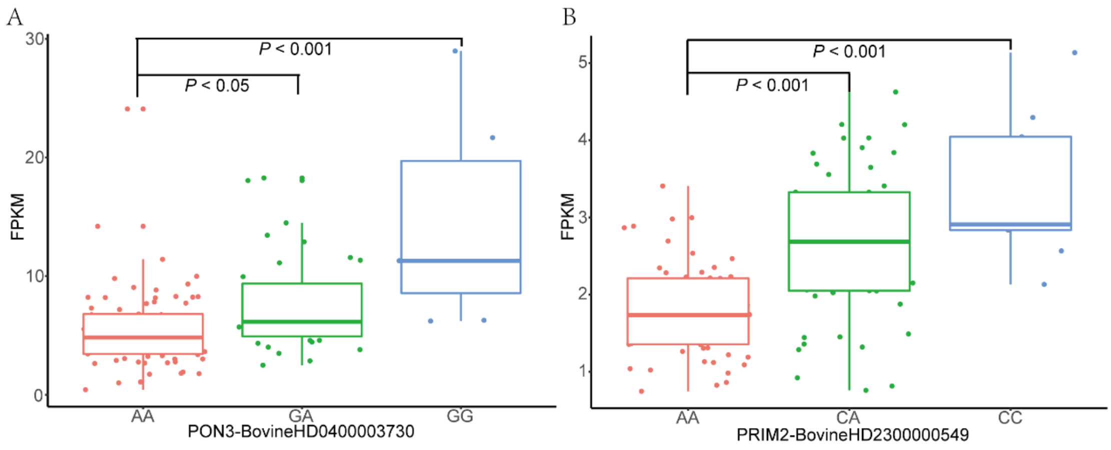

Figure S1. A. The boxplot of the most significant SNP and FPKM value of *PON3*. B. The boxplot of the most significant SNP and FPKM value of *PRIM2*.

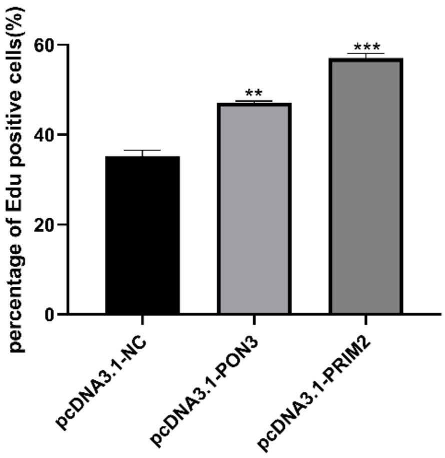

Figure S2. The histogram of EdU-positive cell determination. ** $P < 0.01$,*** $P < 0.001$.
